# Timing Training in Three Children with Diplegic Cerebral Palsy: Short- and Long-Term Effects on Upper-Limb Movement Organization and Functioning

**DOI:** 10.3389/fneur.2014.00038

**Published:** 2014-03-31

**Authors:** Anna-Maria Johansson, Erik Domellöf, Louise Rönnqvist

**Affiliations:** ^1^Department of Psychology, Umeå University, Umeå, Sweden; ^2^Kolbäcken Child Rehabilitation Centre, Umeå, Sweden

**Keywords:** diplegic cerebral palsy, intervention, synchronized metronome training, motor control, kinematic, motor coordination, children

## Abstract

Despite the great need of interventions to maintain and improve motor functions in children with diplegic cerebral palsy (DCP), scientific evaluations of existing training methods are rare. This study aimed to explore individual effects of synchronized metronome training (SMT) on motor timing, spatio-temporal movement organization, and subjective experiences of changes in upper-limb functions in three children with DCP. All children participated in an individualized 4-week/12 session SMT training regime. Measurements before training (Pre), after training (Post1), and at 6 months post completed training (Post2) were made by the applied SMT training equipment, optoelectronic registrations of goal-directed upper-limb movements, and a questionnaire assessing subjective experiences of changes in upper-limb functions and usability. In general, the training regime was shown to have little effect on motor timing. However, some positive changes in spatio-temporal movement organization were found. Two children also reported substantial long-lasting positive changes in subjective experiences of hand/arm functionality in terms of increased movement control and reduced muscle tone. For these children, parallel kinematic findings also indicated smoother and faster movement trajectories that remained at Post2. Although highly individualized, the shown improvements in upper-limb kinematics and subjective experiences of improved functionality of the hands/arms for two of the cases warrant further explorations of SMT outcomes in children with DCP.

## Introduction

The characterization of cerebral palsy (CP) has moved from mainly describing deficits in motor functioning to implicating multiple modalities including sensory, perceptual, and motor problems (1). In addition, therapeutic approaches to CP are beginning to move from an exclusive focus of limiting lower-level motor constraint to also addressing improvements of higher-level derived deficits such as problems with motor planning ability (2, 3). Accordingly, increased attention is being directed toward training methods that encompass improvements in action planning and connections between multiple modalities in children with CP, for example, timing and rhythmicity training or motion interactive games (4, 5).

Assuming that motor performance is mediated by an internal timing mechanism (6, 7), enhanced motor timing is expected to positively affect the performance and planning of motor actions. In line with this notion, repetitive and rhythmic movements have been shown to improve arm paresis following a stroke (8) and induce reorganization of motor networks within the central nervous system (9). Further, interventions based on rhythm perception and production and/or timing and rhythmicity training, such as rhythmic auditory stimulation (RAS), music intonation therapy (MIT), and the Interactive Metronome^®^ (IM), have been reported to improve motor functions in a variety of clinical populations and functions [e.g., Ref. (10–13)]. For example, RAS has been found to re-establish healthy gait dynamics in patients with Parkinson’s disease (14). Of particular relevance to the present study, two recent case studies involving chronic stroke patients (15) and children with hemiplegic CP (HCP) (5) observed positive effects of timing and rhythmicity training in terms of reduced arm impairment, increased functional ability, and better organized goal-directed upper-body movements. Taken together, these reports suggest that timing and rhythmicity training may contribute to increased brain communication, efficiency, and synchrony between brain regions related to motor functions, which leads to improved motor functions and better coordination of movements (16).

The IM is a synchronized metronome training (SMT) device thought to improve the execution of motor programs (17). To this end, the IM apparatus employs a metronome beat to set a rhythm that the participant uses to time motor tasks. A computerized guidance system provides auditory and/or visual feedback to the participant to illustrate the accuracy of synchronization between his/her motor performance and the cueing beat. IM training involves reducing the mean negative synchronization error during normal tracking of a regularly occurring auditory tone metronome beat. Thus, the IM method is targeted at practicing motor planning and timing for enhanced temporal synchronization of movements. As such, the method appears favorable as an intervention for children with CP.

Although the IM and other training regimes seem theoretically promising, there are few studies to date that have used sensitive measurements to evaluate the potential effects of these training methods on performance and/or possible transfer effects to different functions. In this effort, kinematic analysis has been shown to be a promising tool (4) and has been used previously to identify positive short- and long-term effects of IM training in children with relatively mild HCP (5). The aim of the present study was to continue the latter exploration in three children with diplegic CP (DCP), at a more severe level of disability, to investigate whether a similar pattern regarding improved timing ability and potential long-term retention of effects in spatio-temporal movement organization could be observed in these cases following 4 weeks of IM training. Further, a questionnaire aimed at detecting subjective experiences of possible changes in the arms and hands with regard to muscle tone and functionality in daily living activities was administered.

## Materials and Methods

### Participants

Participants included three children with DCP recruited locally through registration records at Kolbäcken Child Rehabilitation Centre in Umeå, Sweden (Table 1). Two participants (case II and III) received upper-limb botulinum toxin treatment but not in close occurrence to their respective individual training and testing period. One participant (case III) received post-surgery (lower limb) physical therapy training in parallel with participation. Informed parental and child consent was obtained, the study was approved by the Umeå Regional Ethical Board and conducted in accordance with the Declaration of Helsinki. In addition, kinematic data from one typically developing (TD) child (girl, 12 years) performing the goal-directed task at one measurement session were collected in order to provide the readers with an example of the task performance differences between DCP and TD shown in Figure 2.

**Table 1:** **Participant demographics, hand and gross motor function, and comorbidities**.

Case	Age (years)	Sex	MACS	GMFCS	Other diagnoses
I	12	F	II	III	ID, autism, epilepsy, visual deficit
II	16	M	IV	IV	ID, dysarthrosis, strabismus, visual deficit
III	13	M	III	IV	ID, CVI, partial epilepsy, strabismus, asthma, scoliosis

*MACS, manual ability classification system; GMFCS, gross motor function classification system; ID, intellectual disability; CVI, cortical visual impairment*.

### Apparatus and procedure

A detailed description of the study design and methods can be found elsewhere (5). All participants underwent 4 weeks of individually adjusted IM training (12 sessions, ~30 min/session), supervised by a trained IM instructor (case I–II: AMJ, case III: assisting physical therapist). Due to the immobility of the legs of the participants, training only involved bilateral and unilateral rhythmic movements of the upper-limbs, with instant auditory feedback (guide sounds) of timing synchronization. Two baseline assessments (2 min clapping to a pre-set beat of 54 bpm with or without guide sounds) were executed at the start of each session and at Pre, Post1 (1 week), and Post2 (6 months). These assessments were used as a measure of individual changes in self-paced and auditory guided timing [deviation in milliseconds (ms) to the auditory signal] and rhythmic performance (variability of motor responses). Variability was measured as the mean deviation from an exact synchronization (regardless of the clap being late or early). Only registered sensor presses (i.e., successful claps) were used in the variability measure. Case II had nearly complete paresis of the right arm/hand, thus, training and baseline assessments were tailored to activate primarily the more functional left side. In addition, case II exhibited severe hypersensitivity toward the auditory presented guide sounds, resulting in spasticity and inability to perform the timing training. Consequently, guide sounds were not introduced until the later stages of the training when case II showed greater audio tolerance. Due to the varying abilities of the children they successfully completed different numbers of repetitions within their individualized IM training. In total, case I completed 13 011, case II 7 746, and case III 6 692 repetitions within their 12 sessions of IM training.

**Figure 1:**
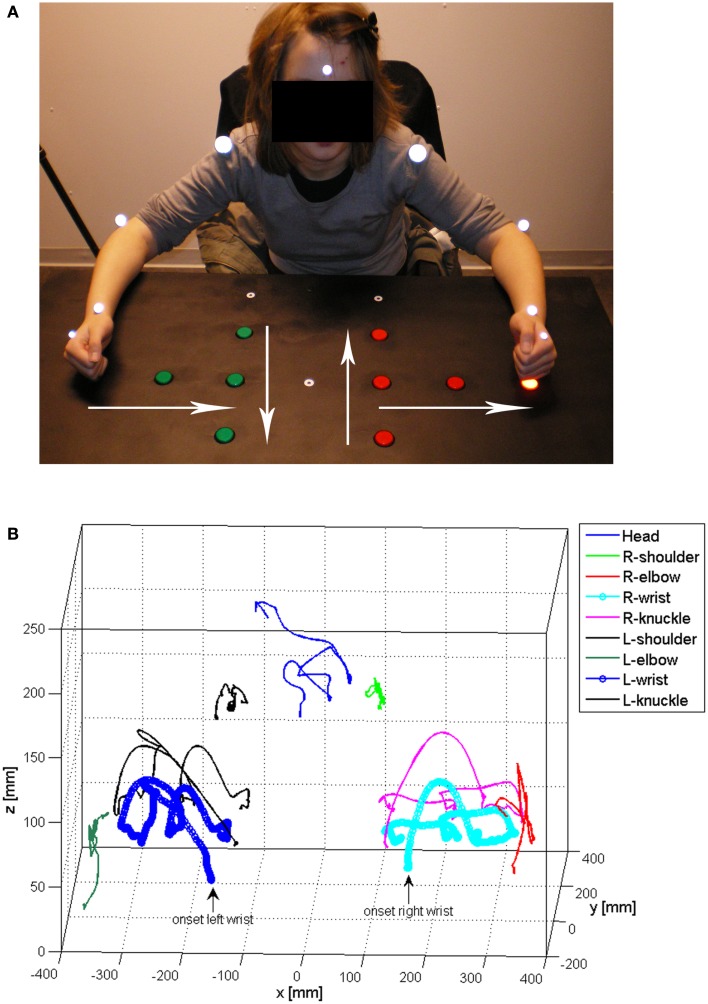
**Photo illustrating the experiment condition and the marker set-up (A), and an example of 3D movement registration (displacement of the corresponding markers) during (bimanual) light-switch task performance (pressing three light-switch buttons in a sequential, side-to-center order) (B)**. Starting positions of the hands are denoted by the small white circles at the lower end of the table in 1A and the white arrows denote the four directions studied (bottom–top, top–bottom, side–center, center–side).

Three-dimensional (3D) kinematic recordings (six-camera, ProReflex, Qualisys Inc., Gothenburg, Sweden) of goal-directed upper-limb movements (pressing three light-switch buttons in a sequential order with a clenched fist) were made before (Pre) and at two occasions after the 4-week training period; Post1 ~1 week after concluded training and Post2 at 6 months after concluded training. For case I, markers were fixated with skin-friendly adhesive tape to the left and right shoulders (diameter: 29 mm), elbows (diameter: 19 mm), and wrists (diameter: 12 mm). For case II and III, markers were only attached to the preferred side (shoulder, elbow, wrist) and non-active shoulder (see Figures 1A,B, for the experiment condition, full marker set-up, and a matching 3D recording).

**Figure 2:**
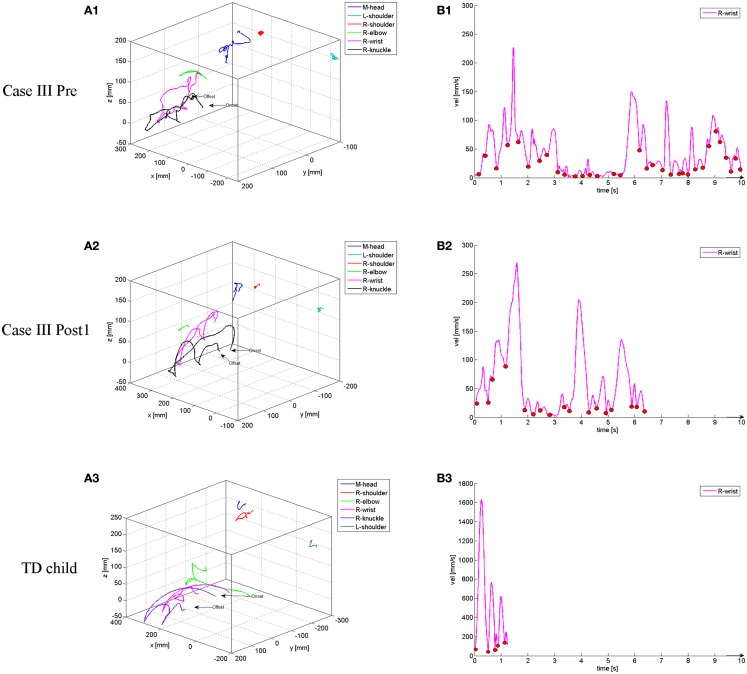
**Examples of 3D movement displacement during unimanual light-switch task performance (starting from resting and sequentially moving to the top button, the middle button, and the bottom button) with corresponding velocity profiles from the wrist marker for Case III at the Pre (A1,B1) and Post1 (A2,B2) occasion, and for a 12-year-old, typically developing (TD) comparison child (A3,B3)**. The small circles in the velocity profiles denote the start/stop of a movement unit (MU) corresponding to 31 MUs/10 s duration in B1, 16 MUs/6.4 s duration in B2, and 4 MUs/1.3 s duration in B3.

The data were sampled at a frequency of 120 Hz/s and the pre-set recording time was individually adjusted based on individual pre-practice and instruction trials that, if possible, were made with both hands. In the unimanual condition, the test paradigm involved performance with either the non-preferred (more affected) or preferred arm–hand (less affected) and with both arms–hands simultaneously in the bimanual condition, corresponding to a total of 36 trials. The participants were instructed to press the light-switch buttons in a sequential order starting on an auditory computer-generated signal. The sequential order was determined by a contra-balanced block design, where the children started from a specific point and pressed the three light-switch buttons starting from the bottom (and moving to the top, “extension”), top (and moving to the bottom, “flexion”), side (and moving inward, “adduction”), or center (and moving outward, “abduction”) with either the right, left, or both hands (see Figure 1A). Due to a severely affected non-preferred side, case II and III only performed the task with the less affected side during testing (12 trials each in total) using the thumb (case II) or index finger (case III) as they were unable to form a clenched fist. The onset and offset of each trial were identified from the 3D movement trajectories (*X, Y*, and *Z* plane) and the tangential velocity of the wrist marker (see Figures 2A,B). On- and off-set were further verified by 2D video recordings that were synchronized with the optoelectronic recording system. The onset of the movement was determined as the frame when the wrist marker had a velocity of 20 mm/s and increased during the following five frames. The offset was defined as the frame when the wrist marker had a velocity of 100 mm/s and increased after the last successful light-switch button press.

Subjective experiences of IM training effects on upper-limb function with regard to muscle tone and functional ability in daily living were collected by means of a questionnaire before training commenced; directly after, and at 3 and 6 months after completed training. Participants were asked to judge changes as; (1) substantially positive, (2) somewhat positive, (3) unchanged, (4) somewhat negative, or (5) substantially negative, with possibility to give open-ended descriptions and examples of any changes in experience.

### Kinematic data analysis

Prior to analyses, the kinematic data were smoothed using a second order 12 Hz Butterworth filter. Extracted parameters from the markers were the cumulative (3D) distance (accumulated movement distance) and the number of movement units (MUs, segmentation of movement trajectories) by use of customized MATLAB (The Mathworks Inc., Boston, MA, USA) scripts. A MU was defined as an acceleration phase followed by a deceleration phase with an accumulated increase or decrease in velocity of at least 20 mm/s and an acceleration or deceleration exceeding 5 mm/s^2^ (18), exemplified in Figure 2B. Further, the duration of each individual task performance was identified and extracted (see Figure 2B). Before statistical analyses, all data were mean valued to one light-switch button press as the number of successful presses varied between trials.

### Statistical analysis

Wilcoxon matched pairs tests with an alpha value of 0.025 were used to analyze differences in kinematic outcomes (based on trial level data) between Pre and Post1, and Pre and Post2. Effect sizes were derived using Pearson’s correlation coefficient for significant results. For case I, no analyses by side were conducted due to an inadequate number of data points (report is thus based on data including both sides). Only significant test statistics and effect sizes of these results are presented. All mean values (*M*), standard deviations (SD), and significant effects are presented in Table 2.

**Table 2:** **Durations, MUs, and 3D distances for the cases presented by occasion**.

	Unimanual								Bimanual							
	Pre		Post1			Post2			Pre		Post1			Post2		
	*M*	SD	D	*M*	SD	D	*M*	SD	*M*	SD	D	*M*	SD	D	*M*	SD
**CASE I**																
Duration (s)	1.4	0.3	+	1.5	0.3	+	1.6	0.7	2.3	0.4	−	2.3	0.4	+	2.4	0.6
MUs (*n*): shoulder	7.7	2.9	+	10.7	6.2	−	7.2	3.8	14.8	4.3	+	17.7	6.4	−	12.1	3.7
MUs (*n*): elbow	5.4	1.8	+	7.4	3.4	−	5.3	2.3	11.2	3.2	+	12.1	3.3	−	9.9	3.1
MUs (*n*): wrist	6.1	2.0	+	6.4	3.3	−	5.4	2.3	11.1	3.6	+	13.0	3.9	−	10.5	2.6
MUs (*n*): head	5.2	2.5	+	7.3	5.3	+	**10.5**	4.3**	8.8	1.9	+	9.8	2.3	+	**12.0**	4.9**
3D distance: shoulder	73	25	+	103	63	+	**77**	22**	108	38	+	**170**	45**	+	118	34
3D distance: elbow	152	57	+	200	134	+	**182**	48**	184	43	+	**238**	39**	+	**235**	42**
3D distance: wrist	205	47	+	248	87	+	232	89	268	52	+	276	70	+	307	54
3D distance: head	58	23	+	**104**	70**	+	**105**	71**	164	67	+	198	43	+	195	46
**CASE II**																
Duration (s)	5.3	1.4	−	4.4	0.4	−	**3.4**	1.3*	
MUs (*n*): shoulder	30.0	10.5	−	28.1	6.0	−	**17.8**	9.8**
MUs (*n*): elbow	32.7	12.7	−	31.7	12.4	−	**18.0**	11.7*
MUs (*n*): wrist	25.0	7.6	−	22.5	7.1	−	**14.3**	9.3**
MUs (*n*): head	19.7	7.5	+	22.4	5.6	+	22.8	17.5
3D distance: shoulder	96	16	+	118	52	−	92	49
3D distance: elbow	234	49	+	328	177	−	187	107
3D distance: wrist	328	57	+	392	140	−	290	181
3D distance: head	373	96	−	245	178	−	**154**	90*
**CASE III**																
Duration (s)	5.5	0.8	−	3.2	0.4	−	**3.1**	0.6*
MUs (*n*): shoulder	58.8	31.7	−	**23.1**	8.4**	−	**22.7**	6.2**
MUs (*n*): elbow	47.9	27.8	−	**24.8**	9.3*	−	**19.7**	4.7*
MUs (*n*): wrist	31.5	14.4	−	**13.0**	5.5**	−	**12.4**	2.8**
MUs (*n*): head	49.1	19.8	−	**18.5**	7.9**	−	**23.0**	4.7**
3D distance: shoulder	76	29	−	73	23	−	42	17
3D distance: elbow	103	21	+	114	83	−	76	22
3D distance: wrist	152	41	+	184	29	+	161	61
3D distance: head	108	29	−	103	42	−	90	50								

**M*, mean; SD, standard deviation; D, direction of change relative to the pre-test (+, increase; −, reduction); s, seconds; MU, mean number of movement units*.

*Significant differences relative to the pre-test occasion are indicated in bold*.

*p < 0.025, **p < 0.01.

## Results

### Case I

#### Training outcomes

**Figure 3:**
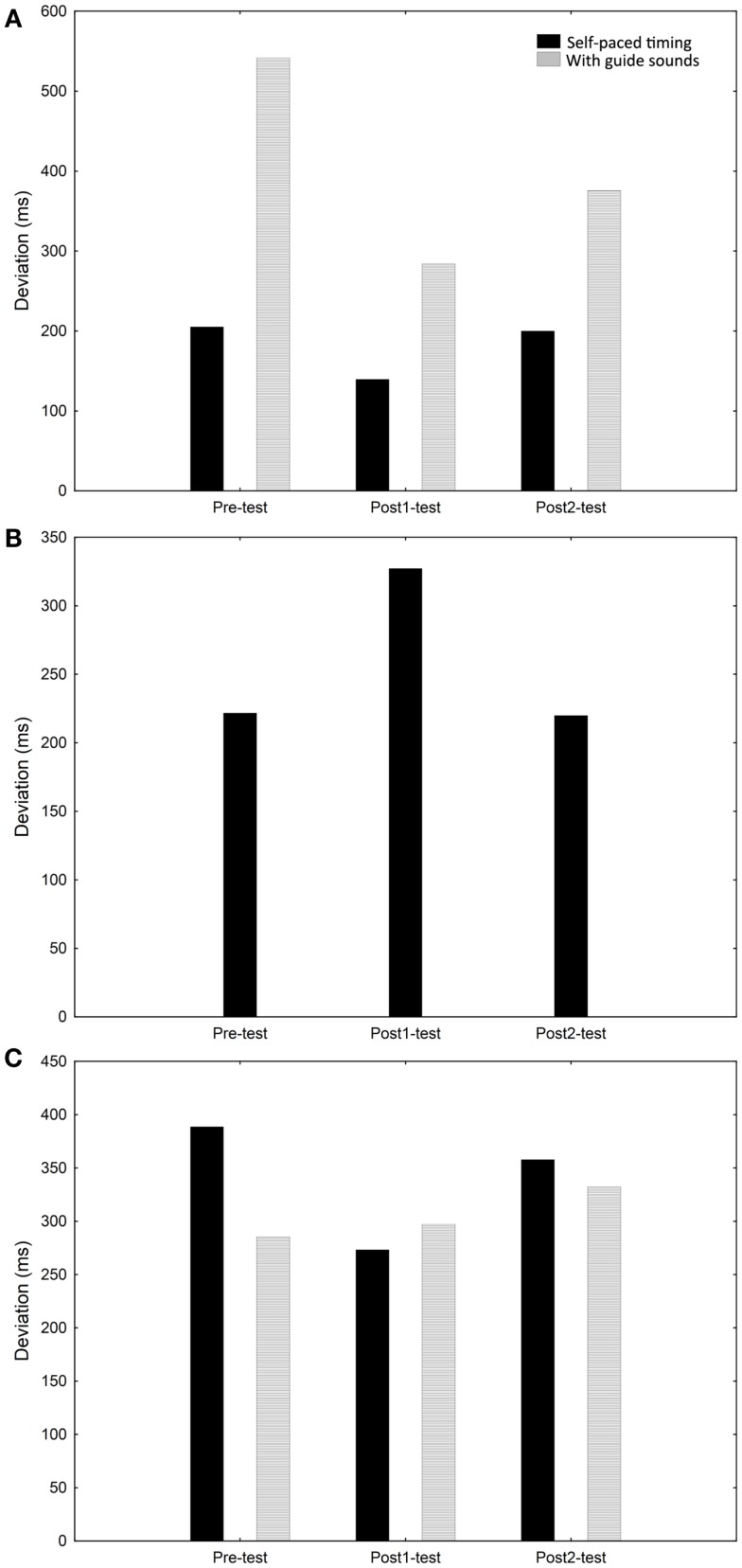
**Rhythmic and timing performance (self-paced and with guide sounds) for case I (A), case II (B), and case III (C) at the Pre, Post1, and Post2 occasions**.

Case I showed a modest improvement in self-paced timing ability (mean timing deviation from exact synchronization without guide sounds) from Pre to Post1 and a more pronounced improvement when guide sounds were included. The variability was lower with guide sounds (millisecond variability; Pre = 151; Post1 = 94; Post2 = 135) than without (millisecond variability; Pre = 542; Post1 = 284; Post2 = 375). The timing ability was not substantially changed from Post1 to Post2 (see Figure 3A).

#### Unimanual condition

A twofold increase in the number of MUs of the head at Post2 compared with Pre (*T* = 3, *p* < 0.01, *r* = 0.10) was shown. The 3D distance of the shoulder increased significantly between the Pre and Post2 occasion (*T* = 4, *p* < 0.01, *r* = 0.51). Similarly, the 3D distance of the elbow increased between Pre and Post2 (*T* = 4, *p* < 0.01, *r* = 0.38). Further, the 3D distance of the head increased from Pre to Post1 (*T* = 23, *p* = 0.01, *r* = 0.12) and Pre and Post2 (*T* = 0, *p* < 0.01, *r* = 0.38). See Table 2.

#### Bimanual condition

As in the unimanual condition, the number of MUs of the head increased significantly between Pre and Post2 (*T* = 31.5, *p* < 0.01, *r* = −0.04). The 3D distance of the shoulder (*T* = 6, *p* < 0.01, *r* = 0.44) and elbow (*T* = 12, *p* < 0.01, *r* = 0.08) increased significantly between Pre and Post1. Further, the increase in distance of the elbow remained at Post2 (*T* = 23, *p* < 0.01, *r* = 0.04). See Table 2.

#### Subjective experience of changes in arm and hand function

Case I reported no changes in muscle tone or the functionality of the arms and hands after completing IM training.

#### Case I summary

Although with large variability, case I showed a modest improvement in timing ability without guide sounds and a more substantial improvement with guide sounds. Although large, the variability in the timing responses did decrease between Pre and Post1, both in the self-paced and auditory feedback conditions. Some significant changes in kinematics were however shown, mainly from the Pre to Post2 tests. Generally, in both the uni- and bimanual condition the 3D distance increased with decreased variability. Specifically, the 3D distance of the head increased, as did the number of MUs in the unimanual condition. Case I reported no effects of the IM training on daily functionality or muscle tonus.

### Case II

#### Training outcomes

Case II showed no improvement in self-paced timing ability and displayed large variability (millisecond variability; Pre = 150; Post1 = 163; Post2 = 161). The self-paced timing ability was slightly worse at Post1 versus Pre but improved again slightly at Post2 (see Figure 3B).

#### Unimanual condition

As shown in Table 2, a significant reduction in movement duration was apparent between Pre and Post2 (*T* = 3, *p* < 0.025, *r* = 0.16). The number of MUs of the shoulder (*T* = 3, *p* < 0.01, *r* = 0.14), elbow (*T* = 5, *p* < 0.025, *r* = 0.21), and wrist (*T* = 11, *p* < 0.01, *r* = 0.23) was also reduced significantly from Pre to Post2. Regarding the 3D distance of the head, a reduction between Pre and Post2 was apparent (*T* = 0, *p* < 0.025, *r* = 0.17).

#### Subjective experience of changes in arm and hand function

Directly after completing IM training, case II reported a substantial improvement with regard to movement ability of the less affected arm and hand (the trained side). Further, the more affected arm and hand were perceived as having substantially less muscle tone, a somewhat improved usability in leisure activities, and a substantial improvement in movement ability. These changes generally remained at the 3- and 6-month follow-up.

#### Case II summary

Case II showed some variability in timing performance during training and at the Pre, Post1, and Post2 test occasions, while no clear improvement in timing ability was shown. The movement duration decreased and some significant changes in terms of kinematics were also shown for this participant, all emerging at Post2. MUs of the shoulder, elbow, and wrist decreased, as did the relative 3D distance of the head. Interestingly, although no real improvements were shown in timing and the effects on movement kinematics did not emerge until the Post2 test occasion, case II reported substantial improvements in movement ability and muscle tone directly after the training period and, although slightly less substantial, after 6 months. More importantly, these changes allowed case II to engage more in leisure activities and to use the less affected arm in daily living situations.

### Case III

#### Training outcomes

Case III showed marginal improvements in self-paced timing ability but not with guide sounds. The variability was stable over the occasions both with (millisecond variability; Pre = 159; Post1 = 181; Post2 = 174) and without (millisecond variability; Pre = 187; Post1 = 183; Post2 = 169) guide sounds. The changes in timing ability from Pre to Post1 were not maintained at Post2 (see Figure 3C).

#### Unimanual condition

The movement duration was significantly reduced between Pre and Post2 (*T* = 0, *p* < 0.025, *r* = 0.71). The number of MUs was reduced between Pre and Post1 of the shoulder (*T* = 1, *p* < 0.01, *r* = 0.29), elbow (*T* = 3, *p* < 0.025, *r* = 0.59), wrist (*T* = 1, *p* < 0.01, *r* = 0.39), and head (*T* = 1, *p* < 0.01, *r* = 0.21). These reductions in movement segmentation remained at Post2 (shoulder: *T* = 1, *p* < 0.01, *r* = −0.09; elbow: *T* = 3, *p* < 0.025, *r* = −0.49; wrist: *T* = 1, *p* < 0.01, *r* = −0.30; head: *T* = 1, *p* < 0.01, *r* = −0.06). See Table 2.

#### Subjective experience of changes in arm and hand function

Case III reported that the less affected arm and hand had somewhat less tone, somewhat improved usability in dressing, feeding, and leisure activities, and somewhat improved movement ability directly after the IM training had concluded. The largest changes, as reported in the open-ended questions, related to movement control, speed, and motivation to activate the hand and arm. The changes that were reported to remain at the 3- and 6-month follow-up were also mainly related to motivation, movement control, and speed.

#### Case III summary

Case III showed a marginal improvement in timing ability with large millisecond deviation from exact synchronization with the metronome and large variability. In the kinematic task, movement duration decreased gradually between Pre and Post2. In terms of kinematic outcomes, a substantial reduction in the number of movement segmentations between Pre and Post1 was apparent and these remained at Post2. The kinematic results suggest large effects on temporal aspects of movement trajectory while subtle and variable changes could be noted on spatial parameters. Case III experienced some meaningful changes in muscle tone and movement ability, which improved elements of daily living ability. The most persistent changes were reported to be related to motivation, movement control, and speed.

## Discussion

The IM training regime aims at facilitating underlying neural processing capacities to improve the execution of motor programs (17). This case study was aimed at exploring the effects of 4 weeks of IM training on timing and rhythmic ability with the arms and hands, planning and spatio-temporal organization of goal-directed upper-limb movements, and the subjective experience of effects on muscle tone and functional ability in daily living in three children with a DCP diagnosis. Of interest was also to investigate the existence of long-term retention of possible effects. Using the same study design and methods as in the present study, we have previously shown that two children with HCP displayed long-lasting motor learning as manifested by remaining timing ability and significant Pre–Post advances in spatio-temporal movement organization following IM training (5). The interpretations of the results from the current study are, however, less straightforward.

### Synchronized metronome training outcomes

In the present study, all cases showed relatively poor initial timing ability with high variability and either modest, marginal, or no convincing improvements at Post1 and Post2. However, an indication of motor learning was apparent for case I who showed some reduction in variability at Post1, both with and without guide sounds, where also a reduction in the millisecond deviation from exact synchronization with the metronome was apparent. Although these effects did not remain at Post2, the results can be regarded as an indication of motor learning during the active phase of training. Perhaps, a longer training period would be preferable for this case. It is also possible that an increased amount of training that included more repeated activations would have improved the timing ability for case II and III. In our previous study including two children with HCP, a relatively good initial timing ability and an evident and stable improvement in timing, both self-paced and with guide sounds, were shown (5). Other studies have shown similar results at a group level in children with ADHD (19), mixed attentional and motor coordination disorders (12), children with no known disability (10), and in skilled golfers (20). Another possible explanation for the present findings is thus that the IM equipment may fail to detect changes in timing and rhythmicity in cases with severe biomechanical constraints.

In general, the present findings suggest that the efficacy of IM training is dependent upon the severity of the child’s condition and the specific constraints that this imposes. Specific constraints may be located in the interpretation and amount of information derived from sensory input as well as in proprioception and the control over muscle groups needed for successful performance (21). A specific constraint may be found in the emergent timing properties of trajectory control that are requested by the SMT method applied. This is relevant because most movements in IM training should be smooth, continuous, and circular in fashion (e.g., hand clapping with circular motions). In such conditions, timing is a by-product that emerges from the dynamics of trajectory control (22) where inability to produce movements with emergent timing elements may be a specific limitation that might account for the relatively poor timing performance of the participants. Further, sources of constraints may be found in the presence of ID, visual deficits, and the diagnosis of autism, which of course poses special consideration of training and outcomes. As such, the training equipment used in this study may not have an optimal design to meet the specific individual needs in order to maximize its accessibility for the participants.

### Spatial and temporal kinematic properties

Although limited improvements in timing ability were shown for all cases, some significant changes in movement kinematics were found. Most kinematic studies of upper-body motor functions in children with CP have focused on HCP (2, 3, 23–29) and investigations into these abilities in children with DCP are sparse. In the current study, substantial reductions in movement segmentations (MUs) were shown for both case II and III. Smoothness of movement trajectory has been shown to have high test–retest reliability when investigating a reach-and-grasp task in children with varying degrees of CP (30) and can be used as a measure of both biomechanical functionality and motor planning ability (27). For case II and III, little change was detected on spatial properties of the movements, suggesting that the increase in smoothness of the movement trajectories has a more temporal character. An accompanying increase in movement speed was also apparent. Taken together, these findings suggest that case II and III showed improvements in motor control and/or planning ability after the IM training. The effects emerged at Post2 for case II and at Post1 for case III, with remaining effects at Post2 for case III, thus indicating a possible reorganization of movement representations in the motor cortex as an effect of SMT. Case I on the other hand showed pre- to post-test increases in MUs of the head in both the bi- and unimanual condition. This finding could be interpreted as an expression of compensatory strategies by means of increased looking. It could, however, alternatively indicate difficulty attending to the task. At the same time, the 3D distances of the (proximal) shoulders and elbows increased, whereas no significant changes over test occasions were detected for the (distal) wrists regarding both 3D distance and MUs. Thus, augmented head and proximal movements did not seem to affect the more distal reaching strategy in case I, suggesting no alternation of the underlying movement representations (and planning) related to the end-motion trajectories as an effect of SMT.

### Subjective experience of training outcome

Despite that no convincing changes were detected in synchronization ability, substantial subjective immediate and long-term improvements were reported in relation to muscle tone, arm/hand functionality, and usability by case II and III. These effects are in line with the more considerable improvements shown in terms of movement organization for these cases.

### Suitability of IM training in children with DCP

Given the results shown in this case study, the IM training regime appears to be a feasible method for upper-limb timing training in children with DCP. However, it is a poor instrument for detecting changes in rhythmic ability and its accessibility seems to be somewhat limited for children with more severe types of CP. For the children participating in the present study, it is plausible that the repeated activation element, rather than the training of synchronization embedded in IM training, was driving the changes detected. Further, the individual biomechanical constraints and co-occurring sensory–motor, cognitive, and neuropsychiatric diagnoses likely reduced the accessibility to the IM training regime. Thus, when considering timing training for children with DCP, it is recommended that special attention should be given to individual needs and abilities and efforts should be made to improve accessibility. Previous studies reporting positive effects of timing training have mainly investigated clinical cases with unilateral brain lesions such as individuals with chronic hemiparetic stroke (8, 15) and HCP (5). On a speculative note, it is possible that this is due to bimanual timing training facilitating effects from the non-paretic to the paretic side. In the case of bilateral brain lesions (involved in DCP), effects may not be underpinned by a similar bilateral transfer of skill.

### Limitations of the present study

Apart from obvious limitations, such as the cases being few and heterogeneous, there are some additional limitations to the present study that need to be addressed. Firstly, the use of two different instructors for the training sessions could have affected the outcome of the training. Given that the training was carried out in accordance with the manual provided by the IM, however, the effect of the instructor should be minimal. Secondly, the IM device does not allow extraction of data other than overall mean values. Unfortunately, this makes the quantification of deviation from the beat in the timing task less optimal. Thirdly, although the participants had to struggle with the simple light pressing task due to the severity of their CP and performed relatively few trials, there is a possibility that improvements at the post-intervention sessions could be related to increased familiarity/practice with the task itself. With this in mind, the use of multiple pre-tests would have strengthened the present design. Alternatively, to control for such potential learning effects in more functionally adept individuals, cases could be habituated to the task prior to the start of the intervention.

## Concluding Remarks

While the effects of IM training on motor timing were unconvincing, several promising changes in kinematic outcomes and functionality could be observed for two of the cases. This case study highlights the importance of developing accessible and individualized training methods that can accommodate the complexity of function that is always associated with early brain lesions that cause CP. Further, the kinematic outcomes pinpoint the importance of developing sensitive measures that are adjustable to the individual competencies of the child with CP. By adopting such an approach, more refined and systematic evaluations of training programs can be made, allowing a better scientific justification of different therapeutic interventions. Based on the current findings, further research investigating the effects of SMT methods in children with DCP are warranted. In future studies, it would be advisable to use larger samples with a case–control design and a dose–response SMT paradigm to maximize individual effects. Additionally, it would be relevant to study the effects of introducing SMT training at an earlier age.

## Author Contributions

Anna-Maria Johansson contributed in conceptualizing and designing the study, was in charge of the IM training and collected the data, carried out the data analyses and interpreted them, prepared the first draft of the paper, and approved the final draft as submitted. Erik Domellöf contributed in conceptualizing and designing the study, took part in and supervised the data collection, participated in the data analyses and their interpretation, co-wrote, reviewed and revised the manuscript, and approved the final draft as submitted. Louise Rönnqvist contributed in conceptualizing and designing the study, took part in and supervised the data collection, participated in the data analyses and their interpretation, co-wrote, reviewed and revised the manuscript, and approved the final draft as submitted.

## Conflict of Interest Statement

The authors declare that the research was conducted in the absence of any commercial or financial relationships that could be construed as a potential conflict of interest.
